# Projected urban growth in the southeastern USA puts small streams at risk

**DOI:** 10.1371/journal.pone.0222714

**Published:** 2019-10-16

**Authors:** Peter C. Van Metre, Ian R. Waite, Sharon Qi, Barbara Mahler, Adam Terando, Michael Wieczorek, Michael Meador, Paul Bradley, Celeste Journey, Travis Schmidt, Daren Carlisle

**Affiliations:** 1 United States Geological Survey, Austin, Texas, United States of America; 2 United States Geological Survey, Portland, Oregon, United States of America; 3 United States Geological Survey, Raleigh, North Carolina, United States of America; 4 United States Geological Survey, Baltimore, Maryland, United States of America; 5 United States Geological Survey, Reston, Virginia, United States of America; 6 United States Geological Survey, Columbia, South Carolina, United States of America; 7 United States Geological Survey, Fort Collins, Colorado, United States of America; 8 United States Geological Survey, Lawrence, Kansas, United States of America; Tanzania Fisheries Research Institute, UNITED REPUBLIC OF TANZANIA

## Abstract

Future land-use development has the potential to profoundly affect the health of aquatic ecosystems in the coming decades. We developed regression models predicting the loss of sensitive fish (R^2^ = 0.39) and macroinvertebrate (R^2^ = 0.64) taxa as a function of urban and agricultural land uses and applied them to projected urbanization of the rapidly urbanizing Piedmont ecoregion of the southeastern USA for 2030 and 2060. The regression models are based on a 2014 investigation of water quality and ecology of 75 wadeable streams across the region. Based on these projections, stream kilometers experiencing >50% loss of sensitive fish and invertebrate taxa will nearly quadruple to 19,500 and 38,950 km by 2060 (16 and 32% of small stream kilometers in the region), respectively. Uncertainty was assessed using the 20 and 80% probability of urbanization for the land-use projection model and using the 95% confidence intervals for the regression models. Adverse effects on stream health were linked to elevated concentrations of contaminants and nutrients, low dissolved oxygen, and streamflow alteration, all associated with urbanization. The results of this analysis provide a warning of potential risks from future urbanization and perhaps some guidance on how those risks might be mitigated.

## Introduction

Worldwide, urban areas are growing rapidly, even in some regions where population growth has slowed. In the USA, urbanized land increased at about twice the pace of the urban population through the 1990s [1]; growth in urbanized land continues to outpace population growth in the USA in recent decades [2]. Urban land use is projected to expand rapidly in the coming decades in the Southeastern USA, especially the Piedmont region, which includes greater Atlanta, Georgia, and Raleigh-Durham-Greensboro, North Carolina [3, 4]. Based on current rates of urbanization, urban land cover in this region is projected to nearly triple from 2009 to 2060, expanding from 17,800 km^2^ to 40,100–54,800 km^2^. Most of this expansion is expected to be commercial and relatively low-density residential development, characterized as “urban sprawl” [3]. What will the effects of this urbanization be on stream ecosystems?

The first step in evaluating the potential changes in stream ecosystems in response to urbanization is to understand how urbanization affects ecosystems under current conditions. Urbanization has long been recognized as having adverse effects on the chemistry, habitat, and biology of streams, disrupting biological communities and causing loss of sensitive species [5–11]. These effects may be related to increases in the number and concentration of contaminants in stormwater runoff, flashier high flows, higher water temperatures, and bank destabilization and channelization [6]. Losses of biological diversity and changes in assemblage composition in urban streams can be dramatic, impairing ecosystem functioning [5, 12–14]. A recent assessment of water quality and ecology of streams across the Piedmont ecoregion provides the data and understanding needed to evaluate relations between urbanization and ecological conditions in the region [15, 16].

Dietze [17] posed two fundamental questions for sustainability in the face of climate and land-use change: “How are ecosystems and the services they provide going to change in the future?” and “How do human decisions affect this trajectory?” Rapid urbanization is relevant to both questions. Knowledge of the future extent of urbanization is needed by resource managers, urban planners, and conservation organizations in order to plan for, and hopefully mitigate any adverse effects on ecosystems. Urban-growth models are used to make these projections, although long-term land-use change is based on combinations of many human actions and is therefore very difficult to predict [3]. Preferences and policies can alter the direction of urban growth, for example, the “smart growth” initiatives underway in many U.S. cities that encourage high-density development in the urban center and discourage urban sprawl. It is unclear, however, if these will substantially reduce the spatial extent of urban development in a fast-growing region like the southeastern Piedmont in the U.S.

The objective of this study was to evaluate how projected changes in urban land use in the Piedmont ecoregion might affect the biological condition of streams. Our hypothesis is that because urbanization adversely affects the biological integrity of streams, expansion of urban areas will result in a greater number of degraded stream kilometers. Models that link current land use and instream biological condition were developed using data from the U.S. Geological Survey (USGS) Southeast Stream Quality Assessment (SESQA) [15]. These models were applied to projected land use for 2030 and 2060 [3] to predict corresponding changes in instream biological condition. The results are interpreted in the context of statistical relations between urban-associated instream stressors—contaminants, nutrients, flow alteration, and habitat—and biological condition [16].

## Materials and methods

**Fig 1:**
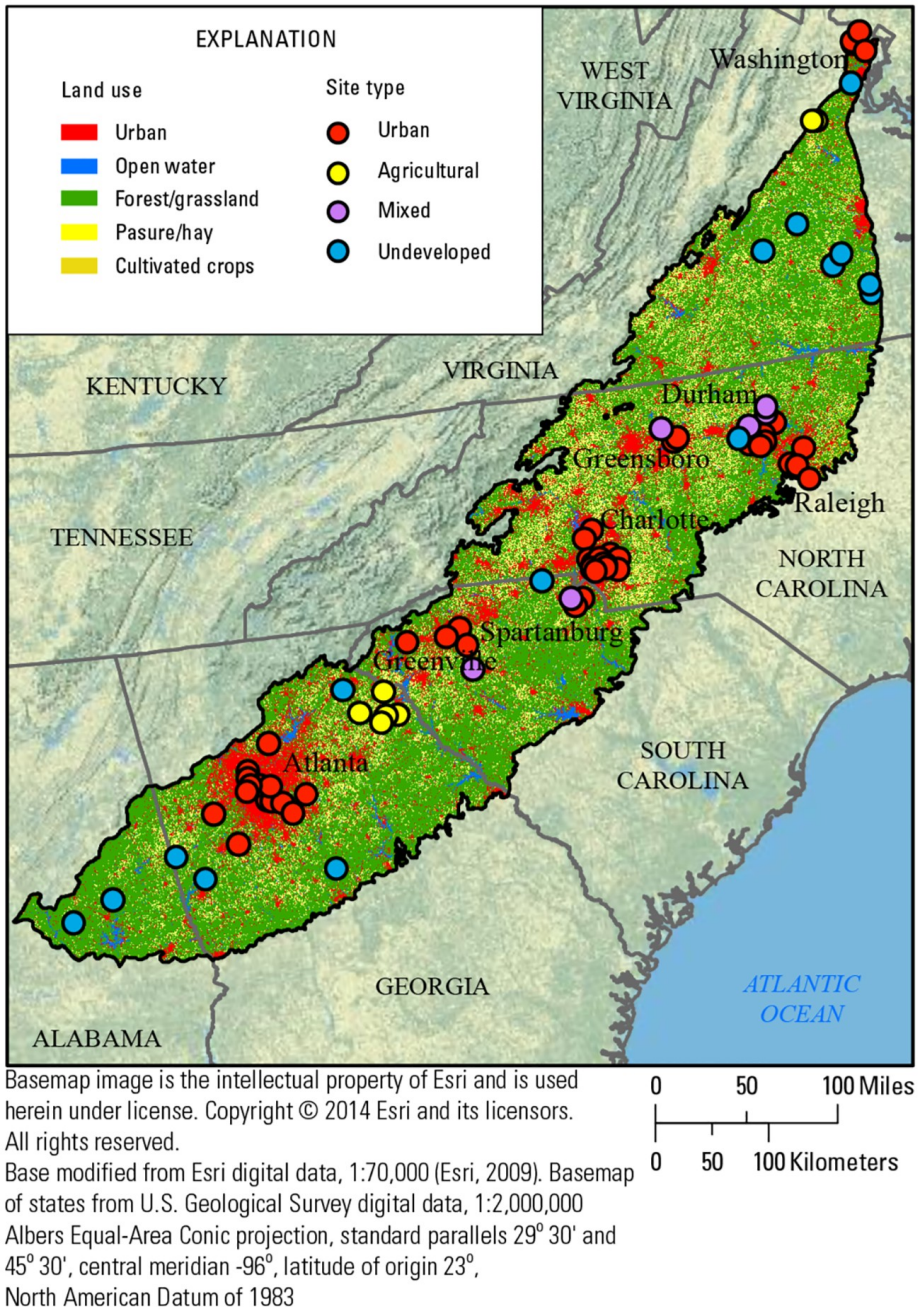
Piedmont ecoregion with Southeast Stream Quality Assessment (SESQA) sampling sites and related land use.

In 2013, the USGS National Water Quality Assessment project (NAWQA) initiated the Regional Stream Quality Assessment (RSQA) (https://webapps.usgs.gov/RSQA/#!/) to characterize the physical, chemical, and biological condition of streams in five major regions of the U.S. and to determine the effects of multiple physical and chemical stressors on associated biological communities [18]. In 2014, the RSQA assessed stream quality in the Piedmont ecoregion in the southeastern USA [15]. The Southeast Stream Quality Assessment (SESQA) sampled 75 perennial wadeable streams across the Piedmont level 3 EPA ecoregion [19], an area of about 166,000 km^2^ covering parts of five states and lying between the Appalachian Mountains to the northwest and the coastal plain to the southeast. (Fig 1; S1 Table). Stream-sampling sites were selected to span a gradient in urban land use in relation to five major metropolitan areas: Atlanta, GA; Greenville and Spartanburg, SC; Charlotte, NC; Raleigh, Durham, and Greensboro, NC; and Washington, DC (Fig 1). These are based on combined statistical areas (CSAs) with the exception that Raleigh-Durham-Chapel Hill, NC, and Greensboro-Winston-Salem, NC, CSAs were combined. Water samples were collected weekly during a 10-week (59 sites; April 7 to June 13, 2014) or 4-week (16 sites; May 13 to June 13, 2104) index period and a wide range of chemicals and physicochemical properties were measured, including nutrients, pesticides, pharmaceuticals, and dissolved oxygen. The sampling index period culminated with collection of bed sediment for chemical analyses and toxicity testing and with an ecological survey of habitat, algae, benthic macroinvertebrates, and fish at each site. Study methods are detailed in Journey et al. [15] and details regarding ecological data and metrics are given in Waite et al. [16]. Full ecological data are available in [20] and ecological and instream stressor metrics are available in [21]. Selected data and metrics also are provided in Supporting Information.

### Land-use projections

Terando et al. [3] simulated changes in urbanization in the Piedmont region on a decadal time step from 2010 through 2060,.modeling the probability that a pixel (60-m pixel resolution) would be urbanized at each time step. The authors classified existing urbanized areas using data from the 2001 National Land Cover Dataset (NLCD) [22] and from local street network information for the years 2000, 2006, 2008, and 2009 from the U.S. Census Bureau’s Topographically Integrated Geographic Encoding and Referencing (TIGER) dataset [23]. Using road network data to assist in the urbanization classification allowed suburban-growth pattern assessments that are more fine-grained and time-sensitive than NLCD ‘Developed’ land-cover based approaches, which can over- and underestimate the low-density growth typical of the Piedmont region [3]. Urbanization was modeled based on a “business as usual” scenario, in which the net effect of growth is in line with that which has occurred in the past. The model simulates patterns of urban expansion that are consistent with observations of past urban growth and transportation networks. Natural and societal controls, such as topographic barriers or regulatory restrictions, are specified in the model parameterization that reduce the likelihood of urbanization.

Variations within urban development (e.g., commercial versus residential) were not modeled, however, “total urban” in the Terando et al.[3] 2009 dataset (Urban2009) correlates very highly (Spearman’s rho>0.92) to other measures of urbanization, including population density, road density, total urban from NLCD2011, and housing density (Table 1). For regions such as the Southeast that depend heavily on cars, the geographic extent of urbanization (which depends not only on population size but also on road networks and the location of often far-flung industrial and commercial centers) may be as relevant to stream condition as population density [3].

**Table 1:** Spearman’s rank correlation coefficients (rho) between Urban2009 and variables retained in BRT models of EPT-H and BIPTAX by Waite et al. (2019) (left columns) and geospatial metrics retained in BRT models presented herein (right columns).

Variable^1^	Urban2009^2^	Variable	Urban2009^2^
Biological metrics		Geospatial	
EPT-H	**-0.779**	HousingDensity2010	**0.958**
BIPTAX	**-0.631**	RoadDensity2014	**0.919**
Nutrients, DO, Temp		PopulationDensity2010	**0.956**
Total Phosphorus	**0.285**	DevelopedLow2011	**0.955**
Total Nitrogen	**0.397**	DevelopedMed2011	**0.905**
Dissolved oxygen, minimum	**-0.332**	DevelopedOpen2011	**0.919**
Habitat		TotalUrban2011	**0.972**
Flow peak interval, mean	**-0.891**	Forest 2011	**-0.885**
Pesticides in water		Soil sand content	-0.102
Number of pesticides det.—W	**0.747**	Base flow index	**-0.353**
Fungicides—P	**0.646**	Dam density 2009	**0.392**
Insecticides -P	**0.724**		
Fipronil and degradates—W	**0.799**		
Sediment Contaminants			
Total PAH TEC (oc)	**0.626**		

^1^Unless noted, variables are medians for the last four weeks of sampling. For pesticides, P indicates variable is from POCIS integrative samplers and W indicates variable is from discrete water samples. For sediment contaminants, (oc) indicates variable is normalized to organic carbon. Stressor metrics are given in [21]; geospatial variable sources and definitions are provided in S3 Table.

^2^ **Bold font** indicates significance at p<0.05.

We used the National Land Cover Data 2011 (NLCD 2011) [24, 25] as the starting point for our land-use mapping and analysis and combined it with the 2009-era urban mapping provided by Terando et al. [3]. This was done to create a full land-cover base that included non-urban classes and upon which future urban expansion could be overlain. Pixels coded as urban in the Terando et al. 2009 dataset were overlain on the NLCD 2011. Pixels from the NLCD that were considered “urban” were those from the Low, Medium, and High Developed classes, excluding the Developed open class [25]. Areas that were coded as urban in either dataset were maintained using a new single urban class for the era 2009–2011 (hereafter, Urban2009). The NCLD 2011 and Terando et al. 2009 urban datasets were very similar in their representation of urbanization, with only minor areas of the original NLCD urban not already coded as urban in Terando et al. 2009. All other classes from the NLCD 2011 (forest, agriculture, etc.) were maintained. This merge of the Terando et al. 2009 urban and NLCD 2011 was termed the NLCD2009m (“modified”). The SESQA watershed boundaries were used to calculate percentages of land use for each of the 75 watersheds from the NLCD2009m, and the land use variables then were used to develop regression models of ecological metrics.

We applied our ecological models to land-use projections for 2030 and 2060, and, for those years, followed Terando et al.’s lead [3] by identifying a pixel as urban at three probability levels to represent uncertainty: a middle estimate (50% probability) and low (20%) and high (80%) estimates. If the probability level is set high (80%), only areas very likely to be urban under the original modeling scenario are included (a more restrictive condition), whereas if the probability level is set low (20%), areas are included as urban that are less likely to be so (a less restrictive condition). Urban land-use projections for the 20, 50, and 80% probability levels from Terando et al. [3] were applied to the NLCD2009m GIS layer in the same way that the Urban2009 was created. This resulted in six GIS layers that together represent the middle estimate and an uncertainty range for 2030 and 2060. Applying urban projections to the NLCD2009m provided updated coverages for other land-use variables for regression models, such as forest, cropland, and pasture. Two assumptions were made in creating these datasets: (1) once a pixel becomes urban it remains urban in future years, and (2) other land-class pixels do not change unless overlain with a new urban pixel (e.g., forest does not convert to cropland or another non-urban class). The latter assumption might seem unwise in some regions, but in the Piedmont, agricultural land use is relatively limited with mostly pasture lands and the primary land use change in recent decades has been conversion of forest and pasture to urban [26, 27].

To model all stream reaches in the region, the NLCD2009m and the six future projections were overlain on the National Hydrography Dataset Version 2 (NHDPlus) [28, 29] polygons associated with all stream segments in the region. A buffer outside of the ecoregion boundary was applied to ensure that streams with headwaters outside of the ecoregion were retained at this step. Each polygon associated with an NHDPlus stream segment represents the land area that drains directly to that segment; that polygon plus all polygons associated with upstream segments thus constitute the total watershed that drains to the downstream end of the segment. For every segment in the Piedmont region, contributing polygons were “accumulated” and their GIS characteristics were determined, providing the full watershed characteristics for all stream segments [30]. Subsequently, segments that fell outside of the Piedmont boundary (the buffered area) were removed to create Piedmont-specific model layers.

Ecological models were based on data from 75 relatively small wadeable streams, with minimum, median, 95^th^, and maximum watershed areas of 5.3, 38, 223, and 491 km^2^, respectively. To ensure that ecological models were applied only to streams similar in size to those streams on which the models are based, all accumulated segments with a total watershed area of more than 250 km^2^ were removed. The remaining watersheds comprise 123,200 km of stream length, 95% of the total stream length in the Piedmont ecoregion (S2 Table).

### Ecological modeling

Boosted Regression Tree (BRT) models were developed for three ecological metrics using a wide variety of current geospatial data for the 75 SESQA stream sampling sites and followed methods described by Waite et al. (2019). Briefly, regression trees fall in the classification and regression-tree (CART) or decision-tree family of techniques [31]. BRT models advance single-classification or regression trees by combining the results of sequentially fit regression trees to reduce predictive error and improve overall performance [32, 33].

The BRT models were developed to evaluate current (2014) ecological conditions relative to the range of geospatial data. Three ecological metrics were modeled using BRT: total macroinvertebrate richness (number of taxa) (RICH); richness of the three dominant sensitive aquatic insect orders Ephemeroptera, Plecoptera, and Trichoptera, minus the less sensitive Hydropsychidae (EPT-H); and richness of benthic invertivore fish as a percentage of all fish taxa (BIPTAX). Although not all of the more detailed geospatial variables are available for future projections, they were used for current conditions to provide insight into those factors most affecting stream ecology and determine whether urban land use can be reasonably approximated by the “total urban” variable available for projected years. Variables tested included various measures of urban development (e.g., housing density, road density and low, medium, and high density urban development) and several landscape and hydrologic variables (e.g., soil sand content, baseflow index). BRT models were run using the *gbm* library in R (version 3.4.3; R Project for Statistical Computing, Vienna, Austria) and code from Elith [33]. Because this code optimizes the number of trees run in each model, the number of trees varies for each model. We reduced explanatory variables in each final BRT model by using a combination of variable importance (VI) scores and evaluation of interactions and partial dependency responses to minimize overfitting.

Only general land-use class variables are available for the 2030 and 2060 projections (e.g., urban, forest, pasture). Given the simplified list of variables available, we chose to use multiple linear regression (MLR) models to project the ecological metrics based on forecasts of land use. These projections rely on the assumption that we can substitute a spatial analysis (models relating ecological condition to current land use) for a temporal change prediction. The underlying assumption is that the relations between land use and ecological responses will not change in the future. The limitations of this assumption and other uncertainties are discussed in the section “Projecting stream condition in response to land-use change”. For dependent variables, two of the three ecological metrics used in BRT modeling were chosen to represent the macroinvertebrate and fish communities: EPT-H and BIPTAX. EPT-H comprises taxa within the orders mayflies, stoneflies, and caddisflies, many of which are considered to be sensitive to environmental disturbance. Benthic invertivore fish are those classified by the EPA as invertivores that prefer benthic habitats [34]. Benthic invertivore fish tend to be smaller endemic species such as darters and sculpin. Twenty-three species of benthic invertivores were found at SESQA sites, all at fewer than one-half of the sites, indicating that most of these species occur infrequently, and thus likely are endemic with small geographic ranges. The southern U.S. contains a relatively large percentage of endemic fish species [35], and endemic species are prone to extinction because of their limited geographic ranges and restricted habitat requirements [35].

Potential independent variables for ecological forecasts were limited to land-use variables projected as described above and two landscape factors, soil permeability and stream baseflow index, that were thought to be important and that were assumed not to change substantially over time. Land-use variables tested in regressions were percent of the watershed in the classes urban (Urban2009), forest (sum of NLCD forest classes), agriculture (sum of NLCD cropland and pasture classes), and wetlands. Assumptions underlying linear regression were examined and reasonably met, as detailed in Supporting Information (S1 Text, Figures A and B in S1 Text).

In addition to evaluations of relations to geospatial variables presented herein, the results from Waite et al.[16] were used to indicate the instream habitat and chemical stressors most likely to influence stream biological condition. Waite et al. developed BRT models for the two metrics used here for land-use-change-based predictions—EPT-H and BIPTAX—and for seven other biological community metrics. Those BRT models related biological community metrics to instream stressors but did not evaluate relations to land use or other GIS variables. Details on the BRT modeling approach are given in Waite et al. [16] and references therein.

## Results and discussion

**Fig 2:**
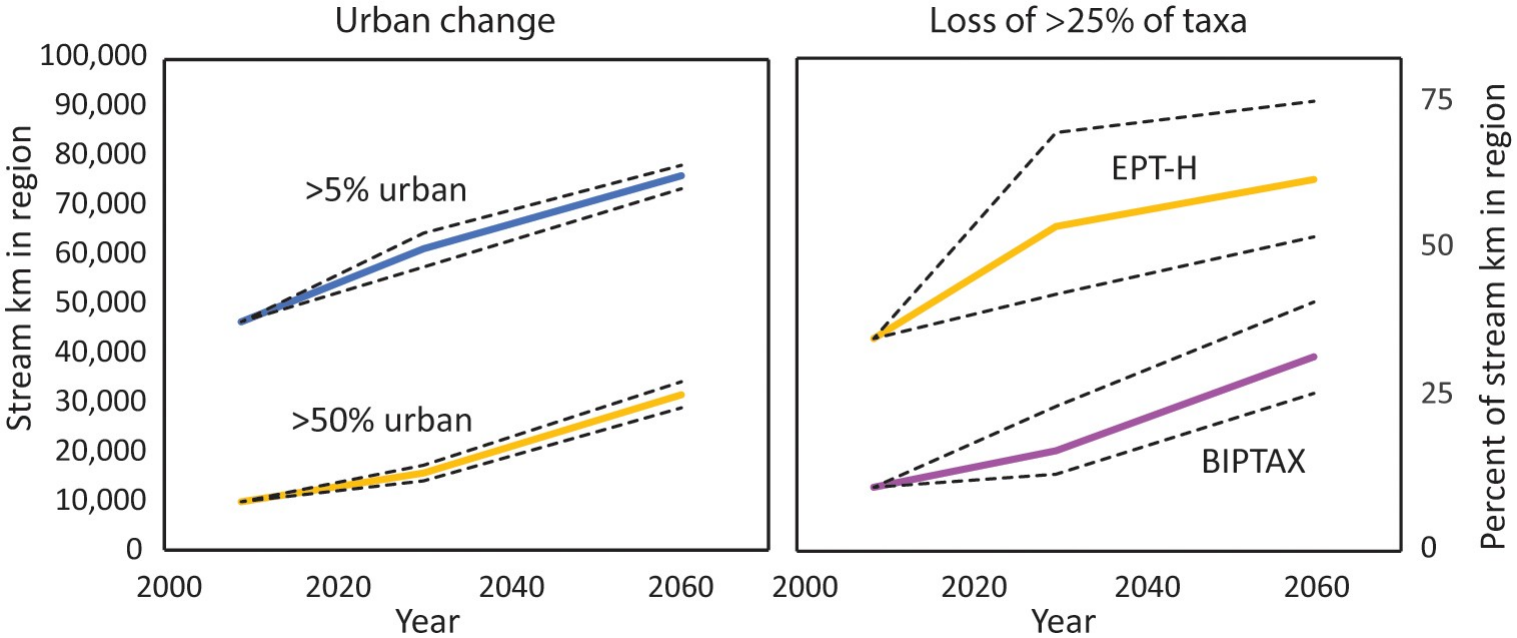
Projected change in urban land use in the Piedmont ecoregion to 2060 (left) and resulting loss of invertebrate (EPT-H) and fish (BIPTAX) taxa (right). Upper, middle, and lower lines for each threshold are 20, 50, and 80% probability of land being urban, respectively. The length and percent of stream kilometers in the region expected to lose >25% of taxa based on land-use change (solid lines); dashed lines represent the 95% confidence interval of the regression.

**Fig 3:**
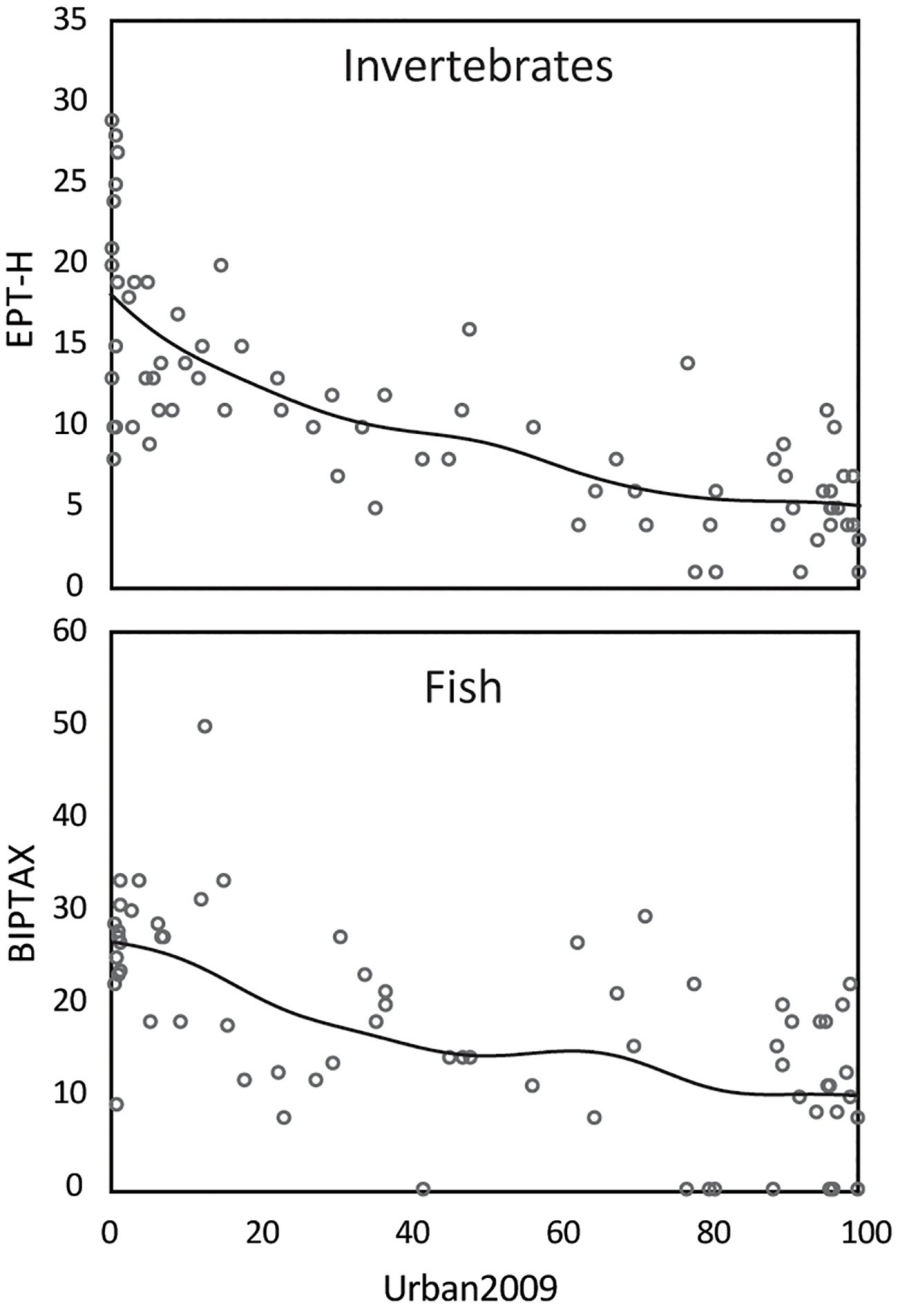
Relations between total urban land use in 2009 and biological metrics. Lines are based on a distance weighted least squares fitting procedure.

As of 2009, 46,700 km of small Piedmont streams (38% of all small streams in the region) had more than 5% urban land use in their watershed and 9,760 km (8%) had more than 50% urban land use. By 2060, 76,200 km of streams, or 62% of all small streams in the region, are expected to have more than 5% urban land use in their watershed (S2 Table) according to the median-probability (of urbanization) projection. The stream kilometers with more than 50% urban land use in their watershed are expected to triple by 2060 to 31,300 km, representing 25% of all stream kilometers (Fig 2), with an uncertainty range of 28,624 to 34,081 km (20 and 80% probability estimates). Urban land use of 50% corresponds to losses of almost one-half of sensitive invertebrate (EPT-H) and one-third of benthic invertivore fish (BIPTAX) taxa (Fig 3). Under the Terrando et al. [3] “business-as-usual” scenario (in which the net effect of growth is in line with that which has occurred in the past), by 2060 the median projection of urban area is expected to increase 165%, from 17,800 km^2^ to 47,500 km^2^, a regional increase roughly equivalent to 4–6 times the current Atlanta metropolitan area. This represents an increase in urban land-use coverage from about 10% to about 30% of the Piedmont ecoregion.

### Current relations between land use and stream ecology

Correlations between urban land use (Urban2009, the total urban class in NLCD2009m) and the sensitive invertebrate and fish metrics for the 75 SESQA sites are strong and highly significant (rho = 0.78 and 0.63, respectively, and p-valuesr<0.001) (Table 1; Fig 3). Substantial losses of invertebrate species are indicated for the more urbanized streams: the least urbanized sites (<5% Urban 2009) have a mean of 60 total taxa and the most urbanized sites (>90% Urban2009) have a mean of 34 taxa. Relative losses for the sensitive invertebrates (EPT-H) are even more pronounced, with means of 18 and 5.1 taxa for least urbanized and the most urbanized sites, respectively (Fig 3). Many species occur frequently at less developed sites but are almost completely absent at more developed sites; these species include mayflies (*Isonychia*, *Caenis*, *Plauditus*), a stonefly (*Perlestra*), and riffle beetles (*Macronychus glabratus*, *Oulimnius nitidulus*) (S4 Table). Conversely, there are some species that occur at most sites regardless of the land use setting, indicating a high level of tolerance to disturbance. Examples include *Cheumatopysche*, a tolerant caddisfly genus in the family Hydropsychidae and three relatively tolerant Dipterans (flies) (*Polypedilum*, *Rheotanytarsus*, *Simulium*).

Relations between fish community metrics and urbanization generally are weaker than for those for invertebrates. For example, the mean number of fish species at the least urbanized sites is 14, and the mean number at the most urbanized sites is 10. The relative effects of urbanization on the benthic invertivores, however, are stronger. The number of benthic invertivore species decreases from a mean of 3.1 at the least urbanized sites to a mean of 1.1 at the most urbanized sites, representing a decrease from 25 to 10% of all fish, respectively. Fish species present at only one or a few relatively unurbanized sites include Blue Ridge sculpin (*Cottus caeruleomentum*), Alabama shiner (*Cyprinella callistia*), Lipstick darter (*Etheostoma chuckwachatte*), and highfin shiner (*Notropis altipinnis*) (S5 Table). The limited ranges of some of these species and their apparent sensitivity to development mean that widespread future development could threaten them with extirpation in the Piedmont.

Boosted regression tree models were developed for three biological community metrics (two invertebrate and one fish) in relation to various geospatial variables (Table 2). The models performed relatively well, with cross-validation R^2^ of 0.47 to 0.63. The models were dominated by several variables related to urban land use or forest (the approximate inverse of urban land use). Urban2009 was included in the variables tested in these models; although it was included in only one final model with a modest variable importance (VI), it correlates very strongly to the other land use variables, such as HousingDensity2010, that are included in the BRT models (Table 1). These models and the very strong correlations between Urban2009 and the important land-use variables the models contain give us confidence that reasonable estimates of future stream condition can be made based on the projection of total urban land use (i.e., projection of Urban2009).

**Table 2:** Comparison of explanatory variables for BRT models for macroinvertebrate and fish metrics for geospatial variables; variables are presented in descending order of variable importance (VI) in each model. See S3 Table for variable sources and definitions.

Macroinvertebrate		Macroinvertebrate		Fish	
Total Richness (RICH) CV R^2^ 0.63	VI	Sensitive inverts (EPT-H) CV R^2^ 0.61	VI	Benthic Invertivores (BIPTAX) CV R^2^ 0.47	VI
HousingDensity2010	54	HousingDensity2010	39	Forest2011	30
DevelopedLow2011	17	Forest2011	28	TotalUrban2011	28
Urban2009	16	RoadDensity2014	19	DevelopedMed2011	14
BaseFlowIndex	13	TotalUrban2011	15	DamDensity2009	11
				Soil Sand Content	9
				DevelopedOpen2011	8

### Projecting stream condition in response to land-use change

One invertebrate (EPT-H) and one fish (BIPTAX) metric were selected to project biological condition in 2030 and 2060 from forecast urban land-use change. These metrics had strong relations to urban land-use indicators (Table 1; Fig 3) and they represent relatively sensitive subsets of invertebrate and fish taxa. The first step was to develop MLR models to relate land-use variables in the NLCD2009m layer to the invertebrate and fish metrics from the 2014 SESQA ecological surveys. NLCD2009m variables include Urban2009 and are consistent with the variables available in the 2030 and 2060 land-use projections. The MLR model for invertebrates had two significant explanatory variables, Urban2009 and AgricultureTotal (sum of cropland and pasture), and performed well with an adjusted R^2^ of 0.64. The regression model for fish had only one significant variable, Urban2009, and a lower adjusted R^2^ of 0.39. The intercept and independent variables in the models had p-values < 0.01. Residuals in both models were normally distributed and did not show any bias in relation to the magnitude of the predicted value (S1 Text).

**Fig 4:**
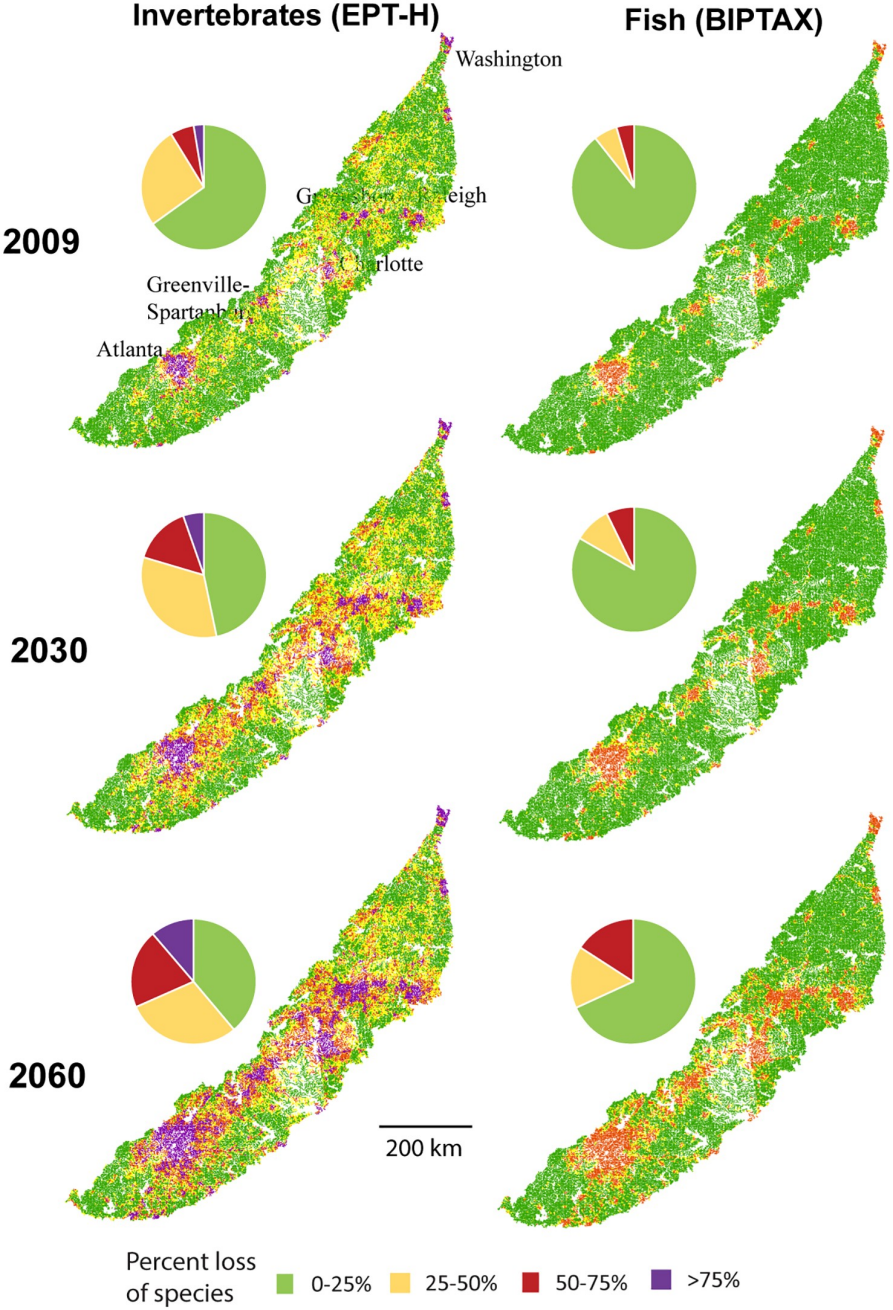
Modeled stream condition for the Piedmont ecoregion for 2009, 2030, and 2060 for number of sensitive invertebrate taxa (EPT-H) and benthic invertivore fish taxa (BIPTAX). Each map shows stream lines in the region colored by the loss of the ecological metric relative to undeveloped sites. Pies show the overall distribution of the metric by stream kilometers in each condition level. Major cities are labeled on the map in the upper left.

The regression models for the invertebrate and fish metrics were applied to the current (2009) and projected (2030 and 2060) land-use conditions for each NHD+ segment in the Piedmont ecoregion that represented a watershed of 250 km^2^ or less. For 2030 and 2060, the regression models were applied to land-use-projection scenarios that correspond to the 20, 50, and 80% probability that pixels would be urban by the given date. Results for the median projection (50% probability of urban) are summarized for each of the 74,603 stream segments in the region by stream kilometers based on quartiles of taxa loss for invertebrates and fish (Fig 4, S6 Table).

The estimated biological condition of streams in the Piedmont ecoregion in 2009 indicates relatively modest impacts of urbanization spatially in terms of species losses. The predicted benthic invertivore fish and sensitive invertebrate taxa for each stream reach was compared to the predicted metric for undeveloped sites, and stream reaches were summarized by quartiles of “loss” of species. For example, the predicted invertebrate metric for a reach with no development was 20.75, so a predicted metric between 10.38 and 15.56 had >25% but <50% loss relative to undeveloped. In 2009, 11% of streams in the region were estimated to have >25% losses of benthic fish (S6 Table). Greater losses are estimated for sensitive invertebrates, with about 35% of streams estimated to have >25% losses and about 9% estimated to have >50% losses relative to undeveloped streams. By 2030, 17% and 53% of stream kilometers are projected to have >25% losses of benthic fish and sensitive invertebrate taxa, respectively. By 2060, about 33% of streams have >25% losses of the fish taxa and 61% have >25% losses of the invertebrate taxa (Figs 2 and 4).

Projected declines in regional stream condition correspond to projected metropolitan expansion and associated infilling of smaller communities along the transportation corridor connecting the major cities. With increasing urbanization, fragmentation of urban “patches” is expected to decrease, with urban areas becoming more connected and with fewer patches that are larger. In contrast, agriculture and forested land cover types are expected to become more fragmented [3]. The increasingly fragmented natural landscape would reduce habitat availability, reduce or eliminate existing natural corridors, and hinder management actions that seek to protect natural systems. By 2060, the Piedmont could have a new, completely connected megalopolis extending from Raleigh, NC to Atlanta, GA (Fig 4), and this continuous urban corridor will have a warmer climate due to both the effects of climate change and the urban heat island. These changes in fragmentation of development, in addition to the overall greater urban land cover, could put further pressure on sensitive invertebrate and fish species, especially those with limited ranges.

There are three major areas of uncertainty in projecting ecological status in streams: uncertainty in models that link ecological condition to land use under current conditions, uncertainty in projecting future land use, and uncertainty in future patterns of chemical use and other urban-related stressors. The uncertainty in the ecological models arises from several factors, including sampling variability and relatively large variation in metrics relative to explanatory variables (e.g., scatter about the lines in Fig 3). Because of these uncertainties, it is not uncommon for models to explain no more than 50–60% of the variance in an ecological metric [36–39].

Uncertainty of the ecological predictions based on the regression models was evaluated by applying the regression models at the upper and lower 95% confidence intervals then computing the numbers of stream kilometers falling in each quartile of taxa loss (Fig 2; dashed lines for graph on the right). On this basis, the uncertainty about the prediction of 75,300 km with >25% loss by 2060 of sensitive invertebrate taxa is from 63,600 to 91,000 km. For benthic fish taxa, the uncertainty about the 2060 prediction of 39,200 km is from 31,700 to 50,600 km (Fig 2).

The uncertainty in projecting future land use is very high because of the difficulty of predicting human actions on the landscape far into the future [40]. This uncertainty is not captured by the 20 and 80% uncertainty in projections modeled here because those projections make the same assumptions as the median projection [3]. Factors, such as weather, that drive internal human migration rates can change over time [41] and events such as technological shocks could force changes to development patterns that would be nearly impossible to predict. The land-use change scenarios presented here therefore should not be viewed as a true prediction of future outcomes [42] but rather as probable outcomes in the absence of fundamental changes to current development patterns. Even under this less restrictive predictive framework based on the trajectory of regional urban growth, the uncertainty for individual locations is high given the complexity of conditions that feed into the decision to develop any particular parcel of land.

Although the methods used in Terando et al. [3] potentially provide a reasonable depiction of suburbanization patterns in the Southeastern U.S., the thematic output of their land-use projection model is coarse in its binary classification (urban or not urban) of future land cover. The binary classification is an artifact of the nature of input urban datasets, as required by the model. The thematic output might limit our ability to make meaningful inferences about future impacts on stream biota, given that the consequences of urbanization will vary with the intensity and nature of urban development [43]. Not only can the intensity and nature of urban development vary within a discrete land-use class, other factors that can affect water quality and ecology can vary, such as soil properties, slope, and channel modifications and impoundments (e.g., [36, 37, 44, 45]). However, these limitations are partly addressed at the watershed scale by representing the intensity of development as the percentage of the watershed that is urban, and, as seen here, a measure of “total” urban at the watershed scale correlates strongly with ecological condition metrics and with several specific measures of urban development intensity (e.g., HousingDensity2010, Table 1).

Yet a third area of uncertainty regards human behavior over time, for example, the continuously evolving use of pesticides [46]. The many reasons behind this evolution include invention of new, more effective chemicals, concerns about effects on the environment, or pest resistance to a particular chemical. As a result, the most commonly used pesticides have changed from inorganic chemicals, such as copper(II)acetoarsenite, used prior to the 1940s, to persistent organochlorine compounds, such as DDT, in the mid-20^th^ century, to the phosphoorganic compounds introduced to replace DDT [47]. More recently, use of pyrethroids, fipronil, and neonicotinoids has increased. Conversely, herbicides such as atrazine and 2,4-D were introduced more than 60 years ago and continue to be heavily used [https://water.usgs.gov/nawqa/pnsp/usage/maps/]. Therefore, although the forecast assumes that chemical use will remain constant for the next 50 years, in reality the types of chemicals that will be used and their toxicity to aquatic biota are not known.

Associated with uncertainty in future land use is uncertainty regarding land-management actions that might affect stream quality. Strategies to manage stormwater runoff, which alters stream-water quality, quantity, temperature, and timing, continue to evolve. Expanded use of green infrastructure, for example, that mimics natural habitats and absorbs excess water has the potential to lessen the impacts of urban land use on stream quality.

### Instream stressors and biology

In addition to other modeling uncertainties, there is uncertainty related to the projection of ecological status based on projected land use. Urban land use, in itself, is not the direct stressor that causes harm to organisms living in streams. Rather, ecological conditions are affected by instream stressors that accompany urbanization, such as streamflow alteration and water-quality degradation. The multi-stressor approach taken by the RSQA studies improves our understanding of which instream stressors most strongly affect biological communities [16, 38, 39, 48] and might provide insight on how some of the effects of future urban development might be mitigated. The BRT models developed for the SESQA by Waite et al. [16] relate instream stressors to biological condition for algae, invertebrate, and fish communities in the Piedmont ecoregion (Table 1; left columns). A BRT model was developed for three metrics for each type of community, for a total of nine models, including models for EPT-H and BIPTAX. All nine models indicated that contaminants were significant instream stressors—in each of the models, at least one of several contaminant metrics that represent eight different pesticide classes or use groups (e.g., fungicides) and two metrics that represent sediment-associated contaminants were retained. Other explanatory variables retained in many models included flow alteration, minimum daytime dissolved oxygen, and either total phosphorus or total nitrogen [16].

### Implications of land-use change

Small streams are critical for freshwater biodiversity and for delivery of ecosystem services but are largely excluded from water-management planning [49]. Under current urban land-use practices, substantial chemical and hydrologic changes occur in small streams in response to urbanization. These changes in Piedmont streams include increased pesticide and nutrient concentrations in water; increased concentrations of PAHs, pesticides, and other hydrophobic contaminants in sediment; flashier, less stable streamflow; lower dissolved oxygen; and higher water temperatures [16]. Most of the stressors included in the nine BRT models of [16] and all of the stressors included in models of EPT-H and BIPTAX (Table 1) are correlated to urban land use. Some of these variables exhibit a relatively linear response to urbanization (e.g., Flow Peak Intervals), whereas others have an exponential response with large increases occurring at higher urban density (e.g., PAHs) (S1 Fig). Awareness of these relations and improved management of these stressors might help mitigate some of the adverse effects of future development on stream health. Reducing contaminant loading, stabilizing flows, and improving aquatic habitat might be achieved, for example, by reductions in chemical use and impervious cover, preservation of riparian habitat, and implementation of green infrastructure and other stormwater management actions.

There are numerous characteristics of urbanization, in addition to those stressors identified by Waite et al. [16], that could be adversely affecting urban stream biota. Freshwater ecosystems are sensitive to both land use and climate change [50–53] including in the Piedmont, as reported by [52] and indicated by the inclusion of urban contaminants, streamflow alteration, and temperature variables in the SESQA stressor models [16]. The SESQA sampling and analysis, however, did not consider a variety of other potential threats to freshwater biodiversity such as harmful algal blooms, microplastic pollution, and engineered nanomaterials [54]. Other potential stressors not measured in SESQA, such as perfluorinated compounds, or not considered in the BRT models, such as non-native species also could be affecting Piedmont stream ecosystems. Additionally, there were variables measured by SESQA but not retained in the BRT models, such as bifenthrin in sediment, imidacloprid in water, and riparian-zone disturbance, which have been shown to adversely affect biological communities in other regions [18, 38, 48]. It is possible that the stressors retained in the BRT models obscured the effects of these or other stressors. Nevertheless, the SESQA study is one of the most comprehensive studies to date to evaluate the effects of multiple stressors on ecology and in relation to the urban landscape. The ecological forecasting analysis presented here provides a warning of potential risks from ongoing and future urbanization and perhaps some guidance on how we might begin to mitigate those risks.

## Supporting information

S1 Table Sampling sites and selected watershed characteristics. (XLSX)

S2 Table Kilometers of stream segments in the Piedmont with >5% and >50% mapped or modeled urban land use in the watershed. (XLSX)

S3 Table Geospatial variable sources and definitions. (XLSX)

S4 Table Invertebrate taxa by site for the Southeast Regional Stream Quality Assessment. (XLSX)

S5 Table Number of fish taxa by site for the Southeast Regional Stream Quality Assessment. (XLSX)

S6 Table Stream kilometers in the Piedmont modeled for three ecological condition metrics and summed by quartiles of taxa loss. (XLSX)

S1 Text Regression analysis. Figure A in S1 Text. Residuals versus predicted values for the EPTR-H and BIPTAX regression models. Red dashed lines represent 95% confidence intervals. Figure B in S1 Text. Normal probability plots of residuals for EPTR-H and BIPTAX regression models.
(DOCX)

S1 Fig Relations between urban land use and in-stream stressors. Graphs of Urban2009 and the 22 significant stressor variables in boosted regression tree (BRT) models developed using data from 75 Piedmont streams. Lines are weighted least squares smoothing (Statistica^®^). Graphs for variables included in BRT models of EPT-H and BIPTAX used in forecasting analysis are shaded tan.
(JPG)
