# Supplementary material for: Projected urban growth in the southeastern USA puts small streams at risk
Source: PLoS One. 2019 Oct 16;14(10):e0222714. doi: 10.1371/journal.pone.0222714 (PMC6795418; doi:10.1371/journal.pone.0222714)
Supplement: S1 Text — Figure A in S1 Text. Residuals versus predicted values for the EPTR-H and BIPTAX regression models. Red dashed lines represent 95% confidence intervals. Figure B in S1 Text. Normal probability plots of residuals for EPTR-H and BIPTAX regression models. (DOCX) [file pone.0222714.s007.docx]

**S1 Text, Regression Analysis**

The principal assumptions of linear regression were evaluated and met for the EPT-H and BIPTAX models. Herein we present evidence that these regression models meet three principle assumptions: linearity and additivity, homoscedasticity, and normality of error distribution. A fourth assumption, independence of errors, is primarily a concern for autocorrelation in time series data. The 75 watersheds sampled here are reasonably assumed to be independent because of their wide geographic distribution and because they are not “nested” within basins.

As can be seen visually in Figure 3 in the body of this paper, the relations between EPT-H and BIPTAX in relation to Urban2009 are reasonably linear. Additional evidence of meeting the first assumption and evidence of homoscedasticity is indicated in plots of residuals versus predicted values (Fig A). For both variables, the residuals are symmetrically distributed around the zero horizontal line and show no trend in magnitude across the range of predictions.

Finally, independence of errors can be tested with a normal probability plot of the residuals (Fig B). For both variables, these graphs indicate that the distribution of the residuals is normal.


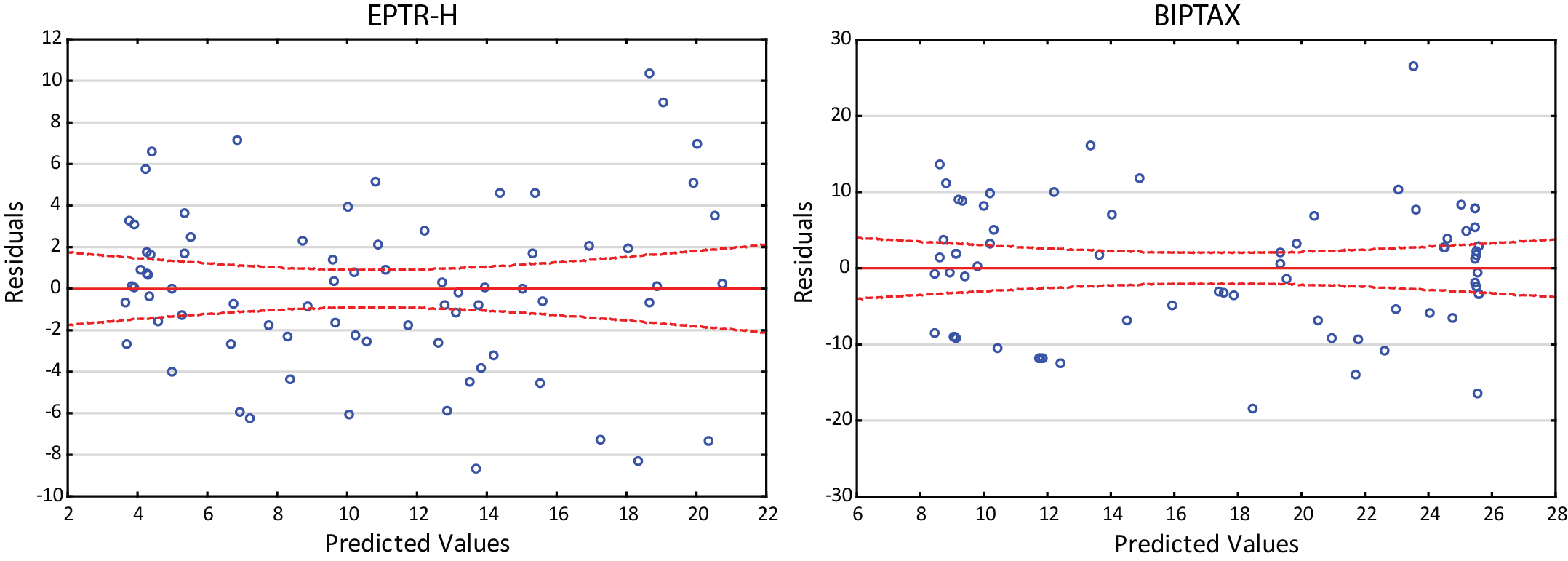


Fig A in S1 Text. Residuals versus predicted values for the EPTR-H and BIPTAX regression models. Red dashed lines represent 95% confidence intervals.


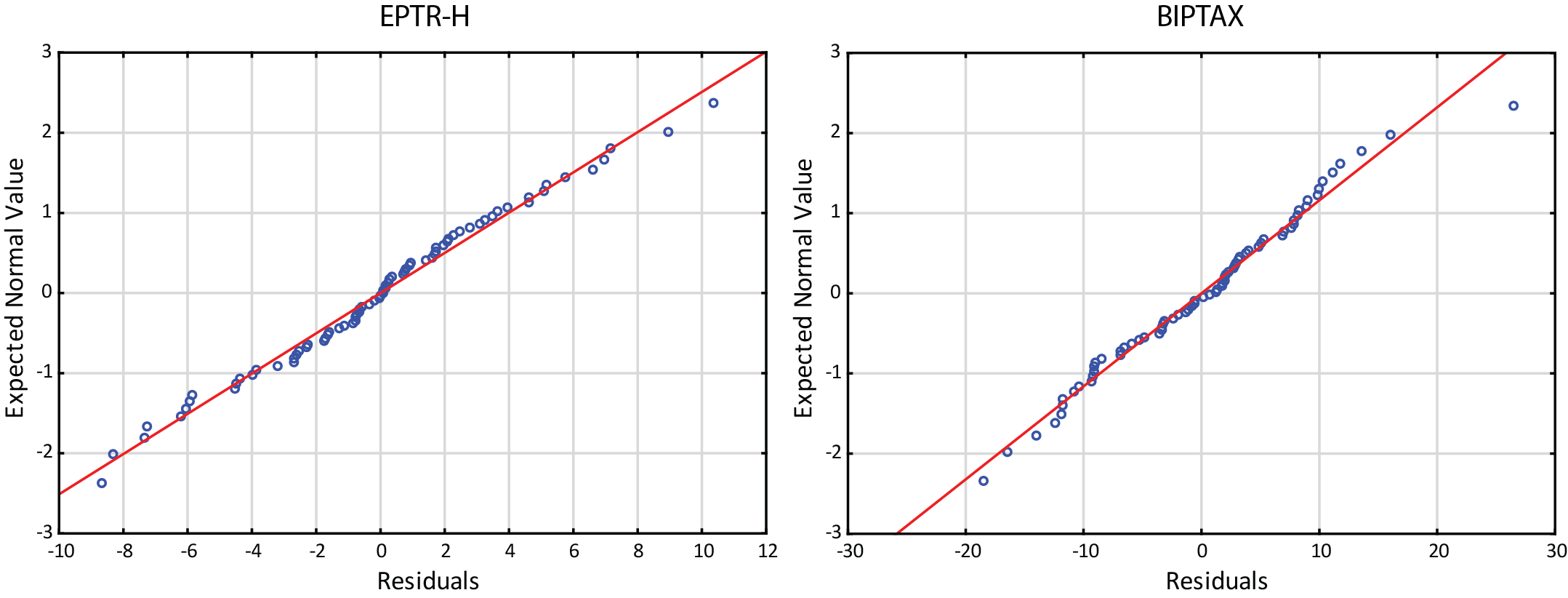


Fig B in S1 Text. Normal probability plots of residuals for EPTR-H and BIPTAX regression models.
