# Supplementary figures and images for: Projected urban growth in the southeastern USA puts small streams at risk
Source: PLoS One. 2019 Oct 16;14(10):e0222714. doi: 10.1371/journal.pone.0222714 (PMC6795418; doi:10.1371/journal.pone.0222714)

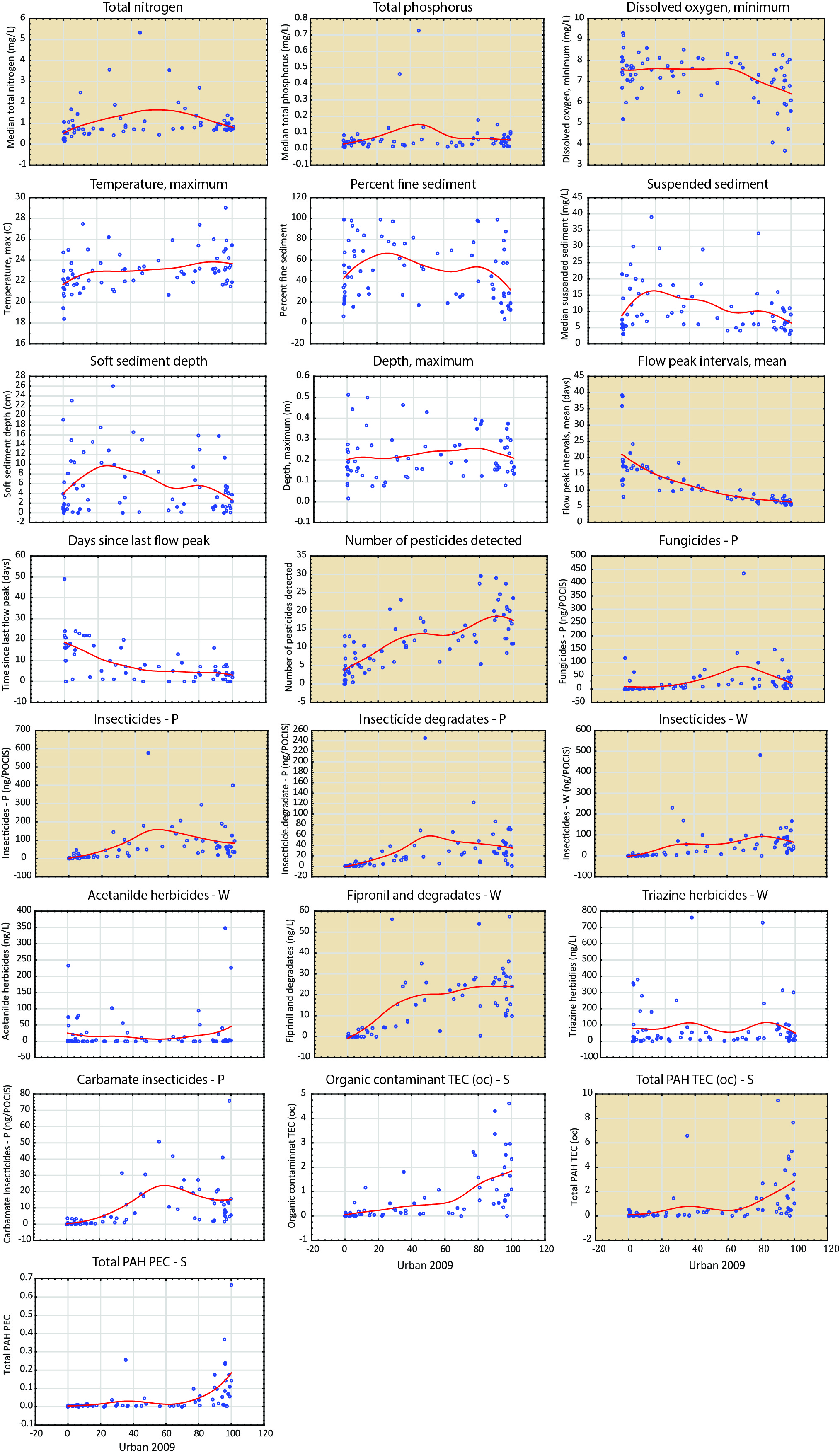

Supplement: S1 Fig — Graphs of Urban2009 and the 22 significant stressor variables in boosted regression tree (BRT) models developed using data from 75 Piedmont streams. Lines are weighted least squares smoothing (Statistica®). Graphs for variables included in BRT models of EPT-H and BIPTAX used in forecasting analysis are shaded tan. (JPG) [file pone.0222714.s008.jpg]
